# Global Analysis of Transcriptome and Translatome Revealed That Coordinated WNT and FGF Regulate the Carapacial Ridge Development of Chinese Soft-Shell Turtle

**DOI:** 10.3390/ijms222212441

**Published:** 2021-11-18

**Authors:** Jun Zhang, Peng Yu, Yang Zhao, Qinyan Zhou, Jiayu Yang, Qingtao Hu, Tiantian Liu, Chuanhe Bao, Shiping Su, Jian-Fang Gui

**Affiliations:** 1 College of Animal Science and Technology, Anhui Agricultural University, Hefei 230036, China; yupeng@ihb.ac.cn (P.Y.); zhaoyang617@stu.ahau.edu.cn (Y.Z.); zhouqingyan@stu.ahau.edu.cn (Q.Z.); yangjiayu@stu.ahau.edu.cn (J.Y.); huqingtao@stu.ahau.edu.cn (Q.H.); liutiantian@stu.ahau.edu.cn (T.L.); baochh1202@ahau.edu.cn (C.B.); sushiping@ahau.edu.cn (S.S.); 2 State Key Laboratory of Freshwater Ecology and Biotechnology, Institute of Hydrobiology, Chinese Academy of Sciences, Wuhan 430072, China; jfgui@ihb.ac.cn; 3 University of Chinese Academy of Sciences, Beijing 100049, China

**Keywords:** Chinese soft-shell turtle, carapacial ridge, WNT, FGF, transcriptome, translatome, turtle shell

## Abstract

The turtle carapace is composed of severely deformed fused dorsal vertebrae, ribs, and bone plates. In particular, the lateral growth in the superficial layer of turtle ribs in the dorsal trunk causes an encapsulation of the scapula and pelvis. The recent study suggested that the carapacial ridge (CR) is a new model of epithelial–mesenchymal transition which is essential for the arrangement of the ribs. Therefore, it is necessary to explore the regulatory mechanism of carapacial ridge development to analyze the formation of the turtle shell. However, the current understanding of the regulatory network underlying turtle carapacial ridge development is poor due to the lack of both systematic gene screening at different carapacial ridge development stages and gene function verification studies. In this study, we obtained genome-wide gene transcription and gene translation profiles using RNA sequencing and ribosome nascent-chain complex mRNA sequencing from carapacial ridge tissues of Chinese soft-shell turtle at different development stages. A correlation analysis of the transcriptome and translatome revealed that there were 129, 670, and 135 codifferentially expressed genes, including homodirection and opposite-direction differentially expressed genes, among three comparison groups, respectively. The pathway enrichment analysis of codifferentially expressed genes from the Kyoto Encyclopedia of Genes and Genomes showed dynamic changes in signaling pathways involved in carapacial ridge development. Especially, the results revealed that the Wnt signaling pathway and MAPK signaling pathway may play important roles in turtle carapacial ridge development. In addition, Wnt and Fgf were expressed during the carapacial ridge development. Furthermore, we discovered that *Wnt5a* regulated carapacial ridge development through the Wnt5a/JNK pathway. Therefore, our studies uncover that the morphogenesis of the turtle carapace might function through the co-operation between conserved WNT and FGF signaling pathways. Consequently, our findings revealed the dynamic signaling pathways acting on the carapacial ridge development of Chinese soft-shell turtle and provided new insights into uncover the molecular mechanism underlying turtle shell morphogenesis.

## 1. Introduction

Turtle is one of the ancient reptiles [1,2,3,4,5]. The turtle shell is a fascinating model in the field of evolutionary and developmental biology because of its unique topological structures [2,3]. The carapace is composed of fused and severely deformed dorsal vertebrae, ribs, and bone plates [4,5]. Especially in the dorsal trunk, turtle ribs grow laterally in the superficial layer and encapsulate the scapula and pelvis, which are markedly different from other amniotic animals [3,5,6]. Thus, the turtle shell is considered to be an evolutionary novelty [4]. The unique structure of a turtle shell has intrigued paleontologists and developmental biologists for nearly two centuries, since the discovery of *Eunotosaurus* opened the door to the study of ancient tortoises [7,8,9]. *Eunotosaurus* was once classified into its own order *incertae sedis* within the subclass Anapsida [10]. In 2010, a phylogenetic study suggested that *Eunotosaurus* was the primitive ancestor of the modern tortoise [11]. According to a taxonomic study, *Eunotosaurus* is a transitional species, which is called “pan Testudines” [11,12]. A *Proganochelys* fossil from 210 million years ago was discovered in Germany in 1887, which had no teeth on the palate and had the same body structure as modern turtles [11]. Although *Proganochelys* differed from modern turtles in many ways, the species already had a fully formed shell. Fortunately, an *Odontochelys* from 220 million years ago was found in China in 2008, which had teeth on the palate and the edge of the mouth [13]. Compared with *Proganochelys*, *Odontochelys* had only a plastron and lacked a carapace, which was consistent with the phenomenon that the ventral shell grows first, followed by the dorsal shell, in the embryo of modern turtle [13,14]. To explain this, the folding theory hypothesizes that sequential development changes that proceeded in a stepwise manner to achieve the turtle-specific body plan [15]. A *Pappochelys* fossil from 240 million years ago was unearthed by German paleontologists in Stuttgart in 2015 [12]. Unfortunately, the fossils of *Pappochelys* were too fragmentary. In 2018, a Chinese paleontologist discovered a new tortoise fossil [16]. *Eorhynchochelys*, a bizarre “chimera”, which played a transitional role in morphology, filled in the gaps of tooth degradation, rib widening, and vertebral reduction, and suggested that turtles probably originated in the land–water environment [16]. Eventually, extensive fossil evidence of the origin of turtles clarified their complete evolutionary sequence. It showed that the unique body structure of turtles was gradually transformed step by step rather than suddenly appearing, and a map of the early evolution of turtles was compiled. The turtle provides an ideal model to study the embryonic development associated with the evolutionary changes in vertebrate morphology. However, only by studying the following two problems can we completely uncover the mystery of the turtle shell. First, what is the molecular mechanism of the development of the tortoise shell? Second, do mutations of genes or gene groups underly the evolution of ancient turtles? Only by understanding the developmental mechanism of the modern tortoise shell can we speculate on the evolution mechanism of ancient tortoises.

The first indication of a departure from representative tetrapod development is the appearance of the carapacial ridge (CR), a ridge along the flank dorsal to the limbs in stage 14 turtle embryos [17]. The CR is a unique tissue of turtle embryos, which comprises overlying thickened epithelial cells encased by mesenchymal cells [4,5,6,18]. In later stages, this ridge elongates anteriorly and posteriorly to became continuous from the base of the tail to the cervical region, and then forms the complete carapace margin [17]. Removing the CR from the snapping turtle *Chelydra serpentina* [17] and Chinese soft-shell turtle *Pelodiscus sinensis* [3] resulted in the ribs crowding into the available adjacent CR. These reports suggest that the CR is a new site of epithelial–mesenchymal transition that is involved in the arrangement of the ribs. Therefore, it is pivotal to explore the regulation mechanism of CR development to gain a deeper understanding of the formation of a turtle shell. Kuraku’s group found that Cellular Retinoic Acid-Binding Protein 1 (*CRABP-1*), Lymphoid enhancer-binding factor 1 (*Lef1*), and Adenomatosis Polyposis Coli Down-Regulated 1 (*APCDD1*) were specifically expressed in the CR of stage-14 *P. sinensis* embryos [5]. They also noted that β-catenin was specifically transferred into the ectodermal cell nuclei in the CR of stage 14 embryos of the Chinese soft-shell turtle [5]. However, the results of this study used a microbead-based screening for mRNAs specifically expressed in the CR, which resulted in 3% of the whole transcriptome being missed by the screening because of the disadvantages of this method [5]. Nagashima and colleagues used electroporation of a dominant-negative form of *Lef1*, which suggested that *Lef1* was likely to be essential in the growth and maintenance of the CR [3]. These reports supported the important role of canonical Wnt signaling pathway in the induction and maintenance of the CR [3,5]. Moreover, Wang annotated the total Wnt genes in the soft-shell turtle and green sea turtle genomes and studied their expression patterns in Chinese soft-shell turtle embryos at Tokita and Kuratani stage 14 (TK14), finding that *Wnt5a* was the only Wnt gene expressed in the turtle CR region [19]. Furthermore, the comparative analysis of different turtle species, including pleurodiran and cryptodiran turtle embryos, hard-shelled turtles and soft-shelled turtles embryos, respectively, implied that the Wnt pathway was the main candidate signaling system involved in the evolutionary origin of the carapace [3,4,20]. In addition, paracrine factors such as fibroblast growth factor (FGF), might be involved in the formation of the shell [21]. However, we still do not fully understand the fundamental factors and regulatory network underlying the CR development of turtles because of the lack of systematic gene screening at different development stages of the CR and gene function verification studies.

Organism morphogenesis during embryonic development is a complex process that requires multiple regulations of patterning and growth direction [22]. Wnt family proteins, including the canonical Wnt/β-catenin pathway, which is involved in regulating cell proliferation and cell fate, and the non-canonical Wnt pathway, which plays roles in developing embryos to converge extension movements, mainly through the planar cell polarity (PCP) pathway [23,24]. PCP is a necessary mechanism underlying coordinated polarized cellular and symmetry-breaking tissue morphogenesis, such as limb patterning, skin hair orientation, and neural tube closure, which is emerging as a crucial conserved mechanism in vertebrates and invertebrates [24]. Existing reports identified a series of proteins that are associated with this noncanonical Wnt signaling pathway in vertebrates, including Frizzled (Fz), Disheveled (Dsh), and Jun-N-terminal kinase (JNK) [25]. Similar to the Wnt binding sites of the Frizzled proteins, *Ror2* also activates the non-canonical Wnt5a/JNK signaling pathway and results in the establishment of PCP in vertebrates [26]. The PCP signaling pathway functions via JNK to control epithelial cell polarity in Drosophila [27]. *Wnt5a* is capable of activating JNK to regulate convergent extension movements in Xenopus [26]. In addition, Wnt5a/JNK and FGF/MAPK are necessary for cell-oriented rearrangements within early limb bud morphogenesis [28]. In the limb mesenchyme, a *Wnt5a* gradient acts as a global cue to instruct the establishment of PCP, and *Wnt5a* also allows FGF signaling to orient PCP [22]. The CR of turtle provides an ideal model to understand multiple regulatory mechanisms of patterning and tissue polarity. However, it is not clear whether the turtle carapace co-opts the conserved PCP pathway to regulate carapace morphogenesis, and the regulatory mechanism of the CR itself remains unclear. Proteins are the actual executors of almost all life activities; however, the sensitivity and accuracy of large-scale proteomic sequencing are not high. Translation regulation is the most important and prominent regulatory link in many levels of the central principle of gene expression [29,30,31,32]. Translatomics is a key technique to determine in the central principle of the flow of gene information from transcripts to functional protein quantification [32]. To clarify the genetic basis of turtle CR development, we selected stages 14, 15, and 16 CR tissue of the Chinese soft-shell turtle as research material and used multiomics correlation of the transcriptome and translatome to systematically analyze the molecular mechanism of the CR development of *P. sinensis* at both the transcription and translation levels. Furthermore, we verified the role of candidate genes in CR development. We revealed dynamic changes in signaling pathways involved in CR development through a correlation analysis of the transcriptome and translatome. Moreover, we revealed that *Wnt5a* regulates CR development through the Wnt5a/JNK pathway. The determination of the molecular mechanism of CR development in Chinese soft-shell turtle has great scientific significance and provides the genetic basis to further reveal the mechanism of formation of the turtle carapace.

## 2. Results

### 2.1. Transcription and Translation Gene Profiles during CR Development

To perform a genome-wide analysis of transcribed and translated genes during the CR development of *P. sinensis*, which is responsible for the turtle carapace morphogenesis, we collected 14th stage (TK14), 15th stage (TK15), and 16th stage (TK16) CR tissues and performed RNA-seq and RNC-mRNA-seq, as shown in Supplementary Figure S1A–C. Three replicates were performed per stage for RNA-seq and one replicate was performed per stage for RNC-mRNA seq. In total, 529,953,950 high-quality (HQ) clean reads were obtained after discarding low-quality reads and adapters reads (see Files S1 and S2). After filtering out rRNA, the reads were compared with the *P. sinensis* reference genome using TopHat2 software, and gene reconstruction was undertaken using Cufflinks (File S3 and S4). After filtering out low-coverage sequences, the assembled genes were compared with the known mRNA reference sequences to identify known RNAs and predict novel genes according to their coding potential. A total of 60,106 transcribed mRNAs were identified among tissues from the three CR stages, including 48,733 known genes and 11,373 novel genes in the transcriptome. Furthermore, 51,080 translated mRNAs were identified among tissues from the three CR stages, of which 41,556 were known genes and 3503 were novel genes in the translatome (File S5).

### 2.2. Dynamic Changes in Signaling Pathways Involved in CR Development

**Figure 1:**
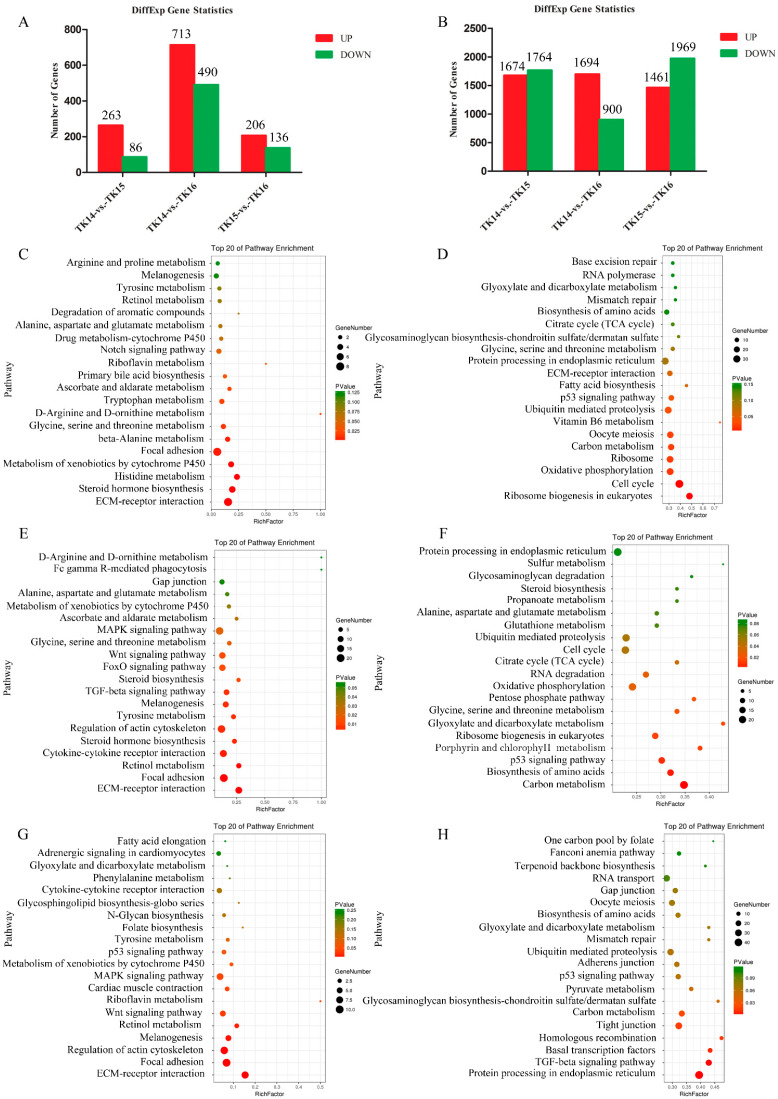
(**A**) DTRGs-TK14 vs. TK15, DTRGs-TK14 vs. TK16, and DTRGs-TK15 vs. TK16. (**B**) DTLGs-TK14 vs. TK15, DTLGs-TK14 vs. TK16, and DTLGs-TK15 vs. TK16. (**C**) Top 20 enriched KEGG pathways of DTRGs-TK14 vs. TK15. (**E**) Top 20 enriched KEGG pathways of DTRGs- TK14 vs. TK16. (**G**) Top 20 enriched KEGG pathways of DTRGs-TK15 vs. TK16. (**D**) Top 20 enriched KEGG pathways of DTLGs-TK14 vs. TK15. (**F**) Top 20 enriched KEGG pathways of DTLGs-TK14 vs. TK16. (**H**) Top 20 enriched KEGG pathways of DTRGs-TK15 vs. TK16.

The FDR and the log2 fold-change (fc) were used to screen for differentially transcribed genes (DTRGs) (FDR < 0.05 and |log2fc| > 1). The results showed that there were 349, 1203, and 342 DTRGs among three comparison groups, respectively. These included 263 upregulated genes and 86 downregulated genes in the TK14 vs. TK15 comparison group; 713 upregulated genes and 490 downregulated genes in the TK14 vs. TK16 comparison group; and 206 upregulated genes and 136 downregulated genes in the TK15 vs. TK16 comparison group (Figure 1A). In addition, FDR and the log2fc were used to screen for differentially translated genes (DTLGs; FDR < 0.05 and |log2 Ratio| > 1). The analysis showed that there were 3438, 2594, and 3430 differential translated genes among three comparison groups, respectively. These included 1674 upregulated genes and 1764 downregulated genes in the TK14 vs. TK15 comparison group; 1694 upregulated genes and 900 downregulated genes in the TK14 vs. TK16 comparison group; and 1461 upregulated genes and 1969 downregulated genes in the TK15 vs. TK16 comparison group (Figure 1B).

To further focus on the signaling pathways that might be involved in regulating CR development, we performed a pathway enrichment analysis from the Kyoto Encyclopedia of Genes and Genomes (KEGG) using DTRGs and DTLGs in the three comparison groups. The results showed that the DTRGs and DTLGs of three comparison groups were enriched for different signaling pathways, respectively. In the TK14 vs. TK15 comparison group, the top 20 enriched pathways of the DTRGs contained the Notch signaling pathway, as shown in Figure 1C. The top 20 enriched pathways of the DTLGs contained the p53 signaling pathway (Figure 1D). In the TK14 vs. TK16 comparison group, the top 20 enriched pathways of the DTRGs contained the TGF-beta signaling pathway, MAPK signaling pathway, Wnt signaling pathway, and FoxO signaling pathway (Figure 1E). The top 20 enriched pathways of the DTLGs contained the p53 signaling pathway (Figure 1F). In the TK15 vs. TK16 comparison group, the top 20 enriched pathways of the DTRGs contained the Wnt signaling pathway, p53 signaling pathway, and MAPK signaling pathway (Figure 1G). The top 20 enriched pathways of the DTLGs contained the p53 signaling pathway and TGF-beta signaling pathway (Figure 1H).

### 2.3. The Wnt Signaling Pathway Might Have a Key Role in Regulating CR Development through a Correlation Analysis of Transcriptomics and Translatomics

**Figure 2:**
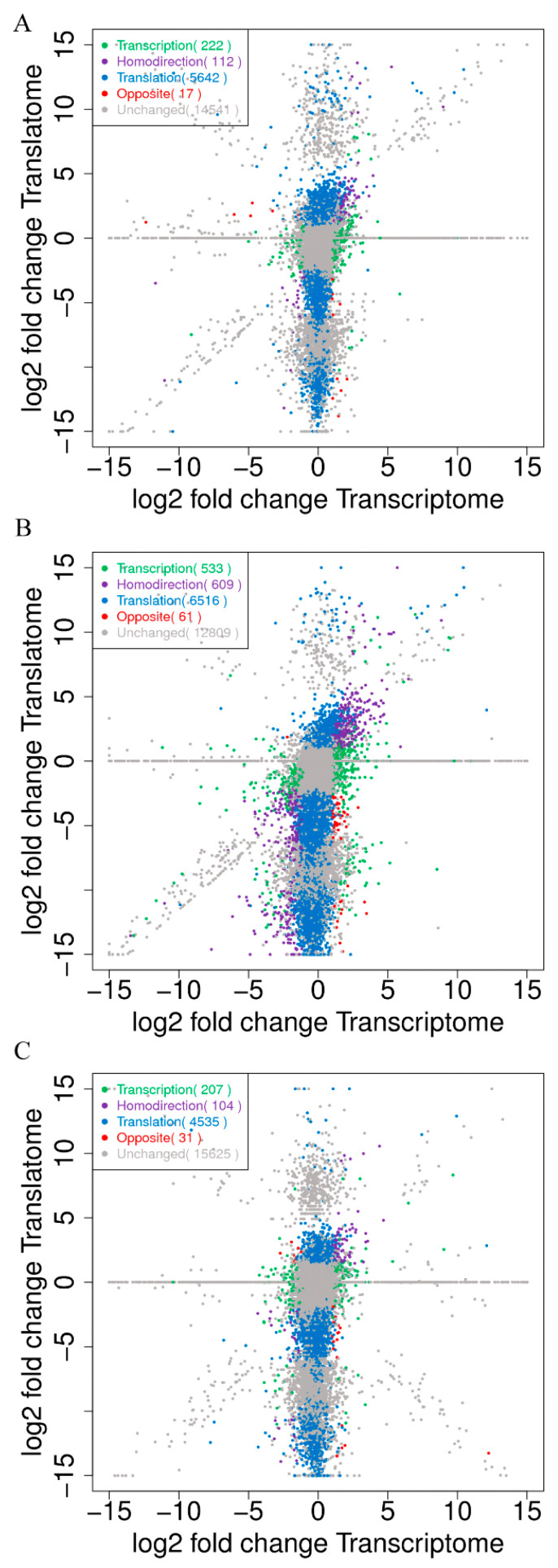
Correlation analysis of the transcriptome and translatome between the TK14, TK15, and TK16 comparison groups of CR tissues. (**A**) Correlation analysis of the transcriptome and translatome of the TK14 vs. TK15 comparison group. (**B**) Correlation analysis of the transcriptome and translatome of the TK14 vs. TK16 comparison group. (**C**) Correlation analysis of the transcriptome and translatome of the TK15 vs. TK16 comparison group. The scatter plot of transcribed genes and translated genes in each comparative group were drawn with the *X*-axis of log2 fold change in the transcriptome and the *Y*-axis of log2 fold change in the translatome. The gray dots represent unchanged genes that were not differentially expressed at either the transcription and translation levels in CR between the comparison groups. The blue dots represent genes that were only differentially translated in CR between the comparison groups. The green dots represent genes that were only differential transcribed in CR between the comparison groups. The purple dots represent homodirection genes, which are differentially transcribed genes and differentially translated genes that were upregulated or downregulated in both analyses. The red dots represent opposite genes, which were differentially transcribed genes and differentially translated genes that were upregulated in one analysis and downregulated in the other.

To perform a correlation analysis of the DTRGs and DTLGs at the level of the transcriptome and translatome among TK14, TK15, and TK16 CR tissues, a scatter plot of the transcribed genes and translated genes in each comparative group was drawn using the *X*-axis of log2 fold change in the transcriptome and the *Y*-axis of log2 fold change in the translatome. In the TK14 vs. TK15 comparison group (Figure 2A), the scatter plot displayed that 70.81% (14,541/20,534) of the transcripts showed no changes at the level of transcription and translation, which are shown by gray dots. A total of 27.48% (5642/20,534) of the transcripts were only differentially expressed at the level of translation and are shown by blue dots; meanwhile, there were 1.08% (222/20,534) of transcripts that were only differentially expressed at the level of transcription, which are shown by green dots. Moreover, 0.55% (112/20,534) the transcripts (purple dots) showed the same direction of differential expression in the level of transcription and translation (termed homodirection), which suggested that the translation efficiency of these transcripts correlated positively with their transcriptional abundance. Interesting, 0.08% (17/20,534) of transcripts (red dots) showed opposite directions of differential expression at the level of transcription and translation, which hinted that the translation efficiency of these transcripts correlated negatively with their transcriptional abundance. In the TK14 vs. TK16 comparison group (Figure 2B), 62.40% (12,809/20,528) of transcripts showed no changes in transcription and translation (gray dots), 31.74% (6516/20,528) of transcripts (blue dots) were only differentially expressed at level of translation, and 2.60% (533/20,534) of transcripts (green dots) were only differentially expressed at the level of transcription. Moreover, 2.97% (609/20,534) of transcripts (purple dots) showed a homodirectional differential expression, and 0.30% (61/20,534) of transcripts (red dots) showed the opposite direction of differentially expression at the level of transcription and translation. In the TK15 vs. TK16 comparison group (Figure 2C), 76.21% (15,625/20,502) of transcripts (gray dots) showed no changes in transcription and translation, 22.12% (4535/20,502) of transcripts (blue dots) were only differentially expressed at the level of translation, while 1.01% (207/20,502) of transcripts (green dots) were only differentially expressed at the level of transcription. Furthermore, 0.51% (104/20,502) of transcripts (purple dots) showed a homodirectional differential expression and 0.15% (31/20,502) of transcripts (red dots) showed the opposite direction of differential expression at the level of transcription and translation. Altogether, the correlation analysis revealed that there were 129, 670, and 135 codifferentially expressed genes including homodirectional differentially expressed and opposite-direction differentially expressed genes at the level of transcription and translation among the three comparison groups (Figure 2A–C), respectively.

**Figure 3:**
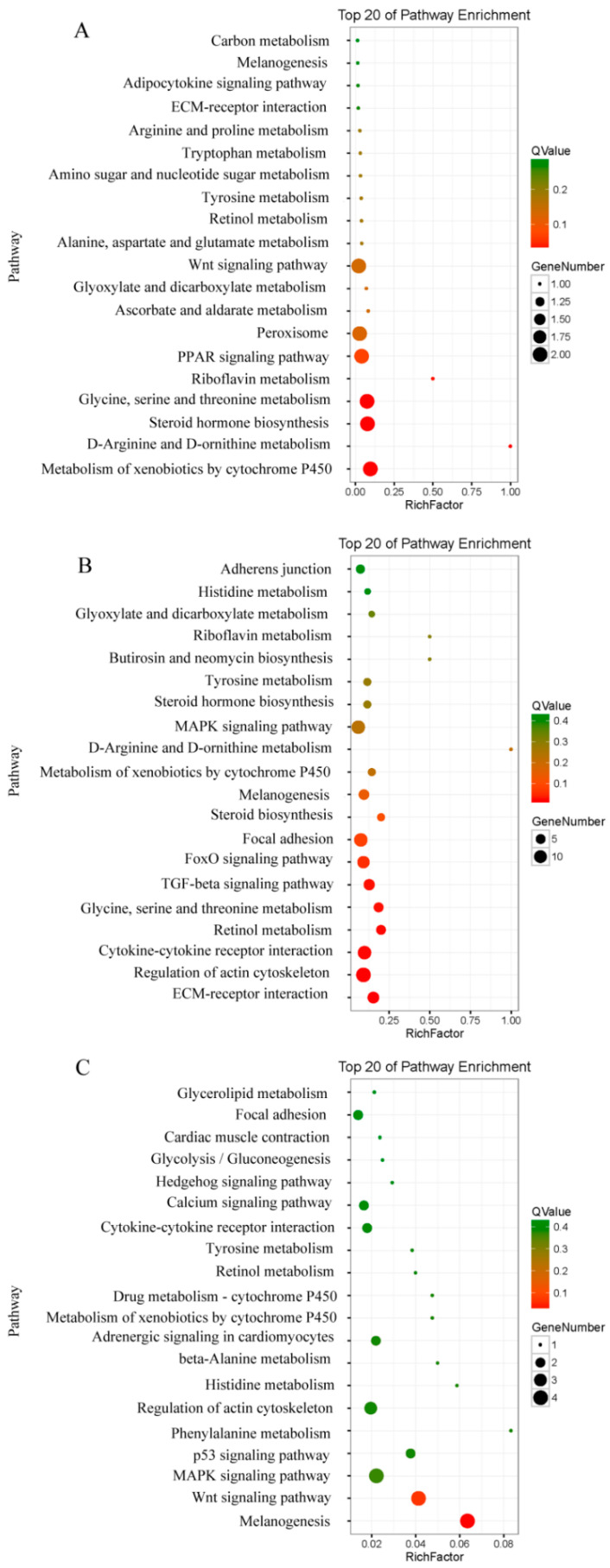
KEGG pathway enrichment analysis of codifferential genes in the three comparison groups. (**A**) The top 20 of enriched pathways in the TK14 vs. TK15 comparison group. (**B**) The top 20 enriched pathways in the TK14 vs. TK16 comparison group. (**C**) The top 20 of enriched pathways in the TK15 vs. TK16 comparison group.

Codifferentially expressed genes at both the transcription and translation levels in the three comparison groups of the three CR stages might play key roles in regulating CR development. Next, we performed a KEGG pathway enrichment analysis using the codifferentially expressed genes in three comparison groups. The results showed that the top 20 enriched pathways of the 128 codifferentially expressed genes in the TK14 vs. TK15 comparison group contained the Wnt signaling pathway, Adipocytokine signaling pathway, and PPAR signaling pathway (Figure 3A). The top 20 enriched pathways of the 670 codifferentially expressed genes in the TK14 vs. TK16 comparison group contained the MAPK signaling pathway, TGF-beta signaling pathway, and FoxO signaling pathway (Figure 3B). The top 20 enriched pathways of the 135 codifferentially expressed genes in the TK15 vs. TK16 comparison group contained the Wnt signaling pathway, MAPK signaling pathway, and p53 signaling pathway (Figure 3C). The pathway enrichment analysis hinted that the Wnt signaling pathway and MAPK signaling pathway might play important roles in the CR development of Chinese soft-shell turtles. In particular, the Wnt signaling pathway might be the key signaling pathway that regulates the CR development of turtle.

### 2.4. WNT and FGF Are Specifically Expressed in CR

**Figure 4:**
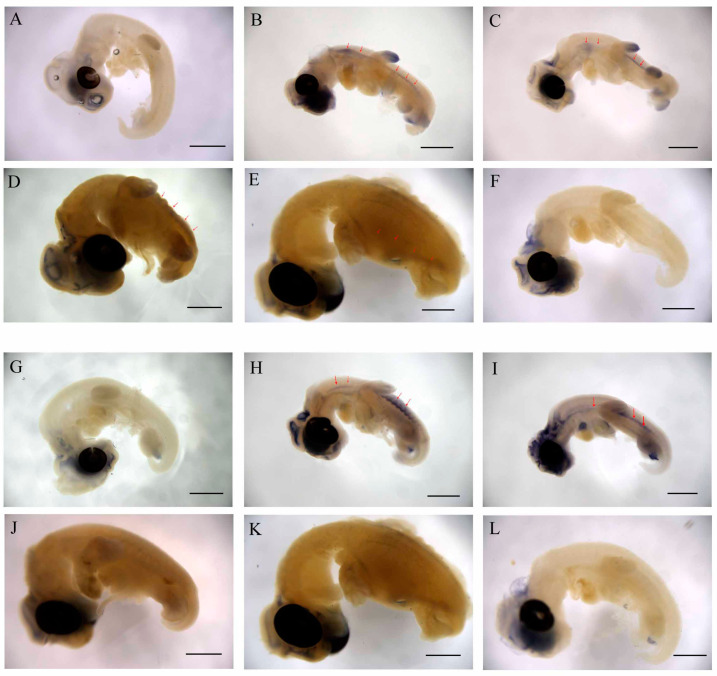
*Wnt5a* and *Fgf10* specific expression in the CR. (**A**–**E**) Expression and location of *Wnt5a* in stages TK13 to TK17 CR of Chinese soft-shell turtle; the red arrow shows the position of *Wnt5a* in the CR. (**F**) Positive probe as a control. (**G**–**K**) Expression and location of *Fgf10* in stages TK13 to TK17 CR of Chinese soft-shell turtle, the red arrow shows the position of *Fgf10* in the CR. (**L**) Positive probe as a control. The scale bar is 2 millimeters.

To further analyze the key candidate genes that regulate the CR development of Chinese soft-shell turtles, we performed whole-mount in situ hybridization (WISH) to analyze the expression localization of *Wnt5a* and *Fgf10* in CR tissue from the 13th stage (TK13) to the 17th stage (TK17) embryos. *Wnt5a* was expressed in the otic vesicle, limb bud, and brain at of TK13 embryos (Figure 4A), while *Wnt5a* expression was not detected in the primitive domain of the CR. Then, *Wnt5a* showed a high expression in CR tissue of TK14 embryos, (red arrows in Figure 4B). Meanwhile, the expression levels of *Wnt5a* in the otic vesicle, limb bud, and brain increased. Furthermore, the expression of *Wnt5a* in the CR remained high in TK15 embryos (Figure 4C). Subsequently, the expression of *Wnt5a* in limb bud and brain decreased significantly in TK16 and TK17 embryos, and the expression of *Wnt5a* in the CR became dispersed in TK16 and TK17 embryos (Figure 4D,E). No expression of *Wnt5a* in the CR was detected in TK14 embryos using a positive probe (Figure 4F). In addition, we found that *Fgf10* was expressed in the otic vesicle, brain, and limb bud in TK13 embryos (Figure 4G), while no *Fgf10* expression was detected in the primitive domain of the CR and lateral plate mesoderm. Subsequently, the expression of *Fgf10* increased in the otic vesicle, brain, and limb bud; moreover, *Fgf10* was expressed intensely in the segmental plate and CR of TK14 embryos (red arrows in Figure 4H). Then, the expression of *Fgf10* was maintained at a high concentration in lateral plate mesoderm and CR in TK15 embryo (Figure 4I). The expression of *Fgf10* in the otic vesicle, brain, limb bud, lateral plate mesoderm, and CR decreased significantly in TK16 and TK17 embryos (Figure 4J,K). We did not detect the expression of *Fgf10* using a positive probe in TK14 embryos (Figure 4L).

### 2.5. Wnts Genes Involved in CR Development Are Not Completely Dependent on mRNA Abundance

To further reveal the roles of the transcription level regulation and translation level regulation of genes in CR development, we compared the number of genes with an expression change in the transcriptome (including transcription, homodirection, and opposite direction) and in the translatome (including translation, homodirection, and opposite direction) in three comparison groups, which are displayed in Figure 2. In the TK14 vs. TK15 comparison group, 349 genes showed expression changes in the transcriptome and 5775 in the translatome (Figure 2A). In the TK14 vs. TK16 comparison group, 1203 genes showed expression changes in the transcriptome and 7189 in the translatome (Figure 2B). In the TK15 vs. TK16 comparison group, 342 genes showed expression changes in the transcriptome and 4677 in the translatome (Figure 2C). The results showed that the number of genes with expression changes in the translatome was significantly higher than the number of genes whose expression changed in the transcriptome in all three comparison groups.

**Figure 5:**
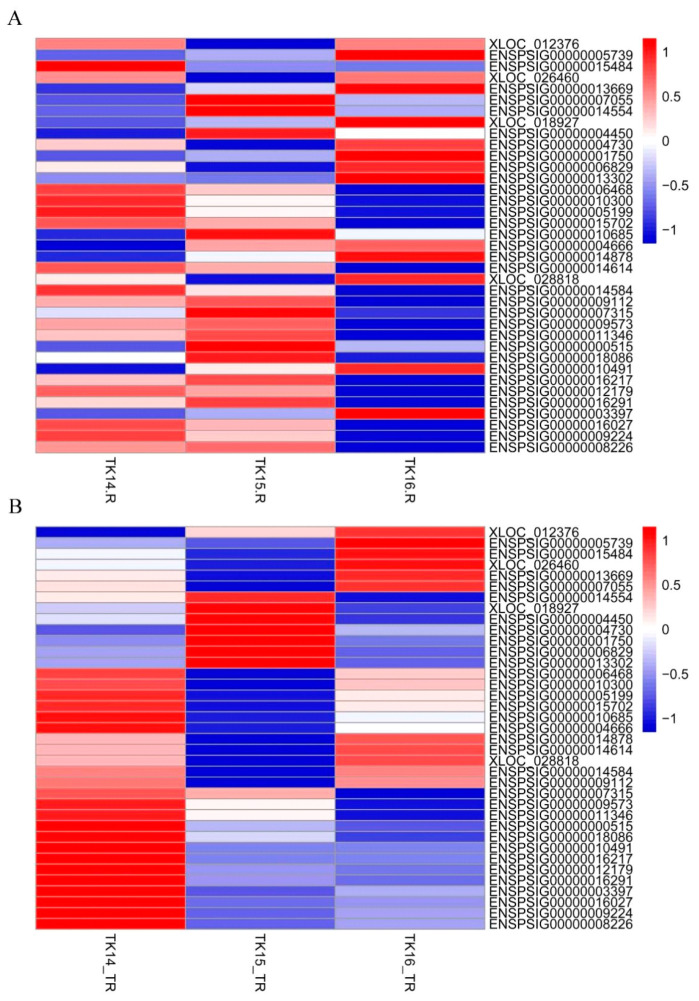
Heatmaps of Wnt members including the mRNA abundance and RNC-mRNA abundance. (**A**) Heatmaps of the mRNA abundance. (**B**) Heatmaps of the RNC-mRNA abundance. The data were depicted as a matrix, in which each row represents one mRNA/RNC-mRNA and each column represents one group. The relative mRNA expression and translated ratio of RNC-mRNA were depicted according the color scale shown on the right. Red represents high relative expression, and blue represents low relative expression; −1, −0.5, 0 and 0.5, 1 are fold changes in the corresponding spectrum. The magnitude of deviation from the median is represented by the color saturation.

**Figure 6:**
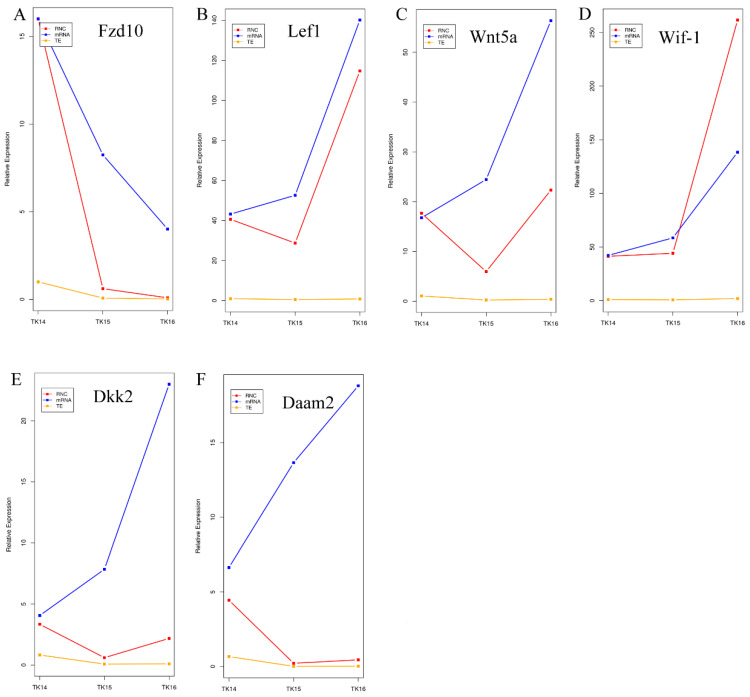
(**A**–**F**) Line plots of transcribed mRNA abundance, translated RNC-mRNA abundance, and translation rate of *Fzd10*, *Lef1*, *Wnt5a*, *Wif-1*, *Dkk2* and *Daam2* in TK14, TK15, and TK16 CR tissues. The red line represents the translated RNC-mRNA abundance, the blue line represents the transcribed mRNA abundance, and the yellow line represents the translation rate.

Furthermore, we drew heat maps of Wnt family members including the mRNA abundance and RNC-mRNA abundance (Figure 5 and File S6), which showed that the transcription and translation trends were inconsistent from TK14 to TK16 for Wnt members, such as *Wnt5a* (ENSPSIG00000003397), *Daam* (*ENSPSIG00000010491*), and *DVL3* (ENSPSIG00000014554). Hence, to further reveal the role of the transcription level regulation and translation level regulation of Wnt members in CR development, a line plot of six codifferential expressed genes was drawn using the mRNA abundance, RNC-mRNA abundance, and the translation ratio from TK14 to TK16 of the CR (Figure 6 and File S7), which were displayed as three different colored lines. The blue lines showed that the mRNA abundance of *Lef1*, *Wnt5a*, *Wif-1*, *Dkk2*, and *Daam2* increased gradually in the CR from stage 14 to stage 16. While the red lines and yellow lines showed that RNC-mRNA abundance and translation ratios of *Lef1*, *Wnt5a*, *Wif-1*, *Dkk2*, and *Daam2* decreased from stage 14 to stage 15. Subsequently, the RNC-mRNA abundance and translation ratio increased from stage 15 to stage 16, as shown in Figure 6B–F. The mRNA abundance of *Fzd10* decreased significantly from stage 14 to stage 16, which was consistent with the RNC-mRNA abundance and translation ratio (Figure 6A). Therefore, the line plot hinted that the regulation of CR development by Wnt members is not completely dependent on mRNA abundance, but also involves translation regulation.

### 2.6. The PCP Signaling Pathway Was Activated during CR Development

**Figure 7:**
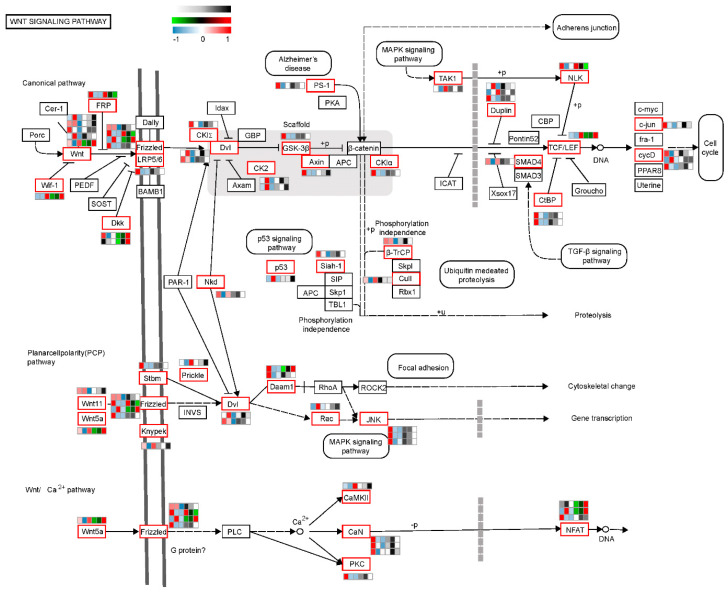
Heatmap of Wnt members at the level of transcription and translation based on the Wnt signaling pathway (ko04310) network map. The genes displayed in the Wnt metabolic pathway heatmap that were differentially expressed in any of the three comparison groups at the transcription level, or differentially expressed genes in any of the three comparison groups at the translational level, are displayed on the heat map. There were a total of 58 genes in the Wnt metabolic pathway heatmap, for which the gene heatmap of the translatome is the three shades on the left and the gene heatmap of the transcriptome is the three shades on the right; heat map homogenization used Zscore. First, if a gene was only differentially expressed at the transcription level, it is displayed in red, black, and green shades at the transcription level and black, gray, and white shades at the translation level. Second, if a gene was only differentially expressed at translation level, it is displayed in red, white, and blue shades at the translation level and black, gray, and white shades at the transcription level. Third, if a gene was differentially expressed at both the transcription and translation levels, it is displayed in red, black, and green shades at transcription level and red, white, and blue shades at translation level.

To further reveal the role of the Wnt signaling pathway’s participation in CR development, we superimposed heat maps of gene expression abundance both at transcription and translation levels among three comparison groups on the Wnt metabolic pathway map (ko04310). The genes were displayed in the Wnt metabolic pathway heat map as follows: (1) Differentially expressed genes in any group of TK14 vs. TK15, TK14 vs. TK16, and TK15 vs. TK16 at the transcriptional level were displayed on the heat map. (2) Differentially expressed genes in any group of TK14 vs. TK15, TK14 vs. TK16, and TK15 vs. TK16 at the translational level were displayed on the heat map. Regarding the heat map presentation: First, if a gene was only differentially expressed at the transcription level, it was displayed in red, black, and green shades at the transcription level and black, gray, and white shades at the translation level. Second, if a gene was only differentially expressed at the translation level, it was displayed in red, white, and blue shades at the the translation level and black, gray, and white shades at the transcription level. Third, if the gene was differentially expressed at both the transcription and translation levels, it was displayed in red, black, and green shades at the transcription level and red, white, and blue shades at the translation level. There were a total of 58 genes added to the Wnt metabolic pathway heat map, in which the gene heat map of the translatome was the three shades on the left and the gene heat map of the transcriptome was the three shades on the right, and the method used for heat map homogenization was the Z-score (Figure 7 and File S8).

The Wnt signaling pathway contains the canonical pathway, the PCR pathway and the Wnt/Ca^2+^ pathway, as shown in Figure 7. The Wnt metabolic pathway heat map showed that Wnt ligands, such as Wnt11, were differentially expressed at transcription level and *Wnt5a* was differentially expressed both at the transcription and translation levels. Moreover, Wnt receptors were differentially expressed at the transcription level, translation level, or both levels, such as Frizzled and LRP5/6. Surprisingly, β-catenin, which is the core member of the canonical Wnt pathway, was not differentially expressed at either the transcription or translation levels. Meanwhile *Lef1*, which encodes the downstream factor of β-catenin, was differentially expressed at both the transcription and translation levels. Furthermore, we found that *Wif-1*, which encoded one of the important antagonists of the Wnt/β-catenin signal pathway, was significantly differentially expressed at both the transcription and translation. *DKK*, which encodes a secreted glycoprotein that plays a negative regulatory role in the canonical pathway by binding to cell surface receptors, was significantly differentially expressed at both the transcription and translation levels. GSK-3β, Axin, and APC form a complex with βcatenin, which reduces the level of free β-catenin in cells and inhibits the Wnt/β-catenin signal pathway. These three genes showed a marked differential expression at the translation level. These results hinted that the canonical pathway might be inhibited during CR development. Paradoxically, the gene encoding the downstream target β-catenin, *Lef-1*, was significantly upregulated at the transcription and translation levels during CR development. The reason might be that β-catenin regulates CR development not through transcriptional and translational regulation, but through the regulation of the transfer of βcatenin to the nucleus to perform its functions.

Interestingly, we found that the PCP pathway was activated during CR development. As shown in Figure 7, *Wnt5a*, *Wnt11*, and *Fz* were differentially expressed at the transcription level, the translation level, or both levels in the PCP pathway, and we also discovered that *Daam1* was differentially expressed at both the transcription and translation levels. Moreover, *Dvl*, *Rac*, and *JNK* in the PCP pathway were differentially expressed at the translation level among the three comparison groups. The results strongly suggested that the Wnt5a/PCP pathway might play important role in regulating CR development in Chinese soft-shell turtle.

### 2.7. Wnt5a Participate in the CR Development through the JNK Pathway

**Figure 8:**
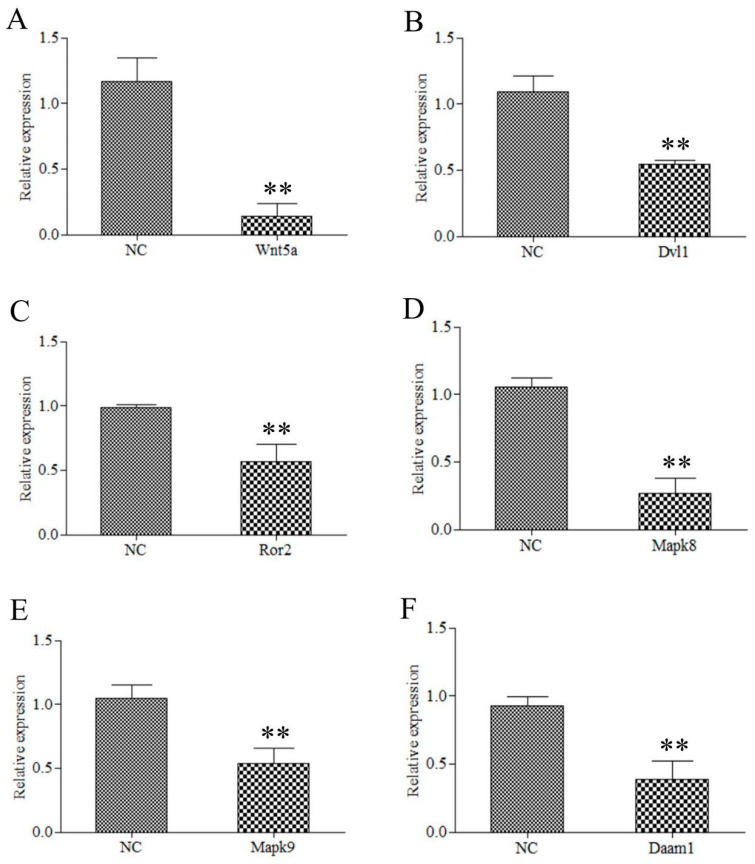
Knockdown of the expression of *Wnt5a* in CR tissues cultured in vitro. (**A**) The expression level of *Wnt5a* in the 5′ Cho-si-*Wnt5a* group was significantly lower than that in NC group after 48 h of 5′ Cho-si-*Wnt5a* interference. (**B**–**F**) The expression levels of *Dvl1*, *Ror2*, *Mapk8*, *Mapk9*, and *Daam1* in the 5′ Cho-si-*Wnt5a* group were significantly lower than those in the NC group after 48 h of 5′ Cho-si-*Wnt5a* interference. ** represents extremely significant difference (*p* < 0.01).

To further reveal the molecular mechanism of *Wnt5a* in the regulation of CR development, we knocked down the expression of *Wnt5a* through siRNA-mediated interference with *Wnt5a* expression in CR tissues cultured in vitro. To test the interference efficiency of 5′ Cho-si-*Wnt5a*, we performed qRT-PCR using primers for *Wnt5a* at 48 h after CR tissues were transfected with 5′ Cho-si-*Wnt5a*. The results showed that the expression level of *Wnt5a* in the 5′ Cho-si-*Wnt5a* group was significantly lower than that in the NC group, as shown in Figure 8A. These results indicated that 5′ Cho-si-*Wnt5a* could effectively knock down the expression of *Wnt5a*. Furthermore, to reveal the effect of the knock down of *Wnt5a* in CR tissues cultured in vitro on the downstream genes of the Wnt signaling pathway, we detected the expression of *Dvl1*, *Ror2*, *Mapk8*, *Mapk9*, and *Damm1* at 48 h after CR tissues were transfected by 5′ Cho-si-*Wnt5a* using qRT-PCR. The results showed that the expression levels of *Dvl1*, *Ror2*, *Mapk8*, *Mapk9*, and *Damm1* in the 5′ Cho-si-*Wnt5a* group were significantly lower than those in the NC group (Figure 8B–F). Therefore, we concluded that *Wnt5a* regulates the CR development of Chinese soft-shell turtle through the Wnt5a/JNK pathway.

### 2.8. The Expression of Wnts during the CR Development by qRT-PCR

**Figure 9:**
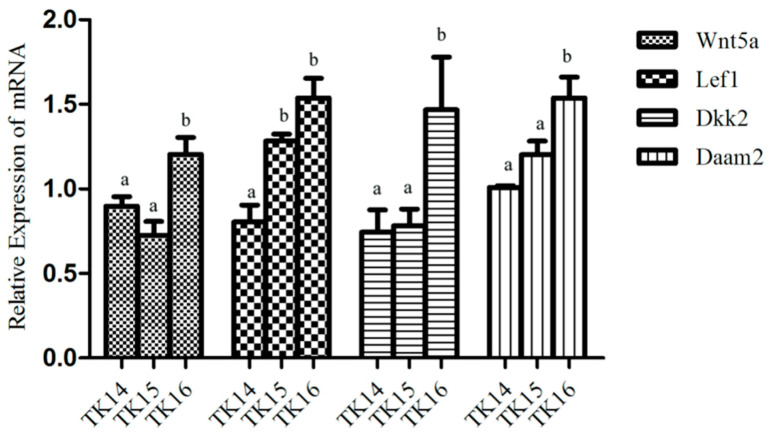
The expression of Wnts in CR tissues from TK14 to TK16 were detected by qRT-PCR. The mean values sharing different superscripts are significant different (*p* < 0.05).

Differentially expressed transcripts of Wnts, including *Wnt5a*, *lef1*, *Dkk2*, and *Daam2* were identified in the RNA-seq data of CR tissues from TK14 to TK16, and their expression trends during CR development were validate by qRT-PCR. The results displayed that the expression abundances of *Wnt5a*, *lef1*, *Dkk2*, and *Daam2* increased gradually in CR tissues from TK14 to TK16, which was consistent with the transcriptome (Figure 9).

## 3. Discussion

The turtle carapace is an example of an evolutionary novelty, the formation mechanism of which is still debated [33]. CR is a turtle-specific transient embryonic tissue that is involved in governing the positioning of the dorsal rib, which serves as a key to investigate the molecular mechanism of turtle carapace morphogenesis [5]. Although research has been performed on the CR for decades, the molecular interactions within CR remain obscure. In this study, we obtained a genome-wide gene transcription and gene translation profile through RNA-seq and RNC-mRNA seq using CR tissues at three stages of Chinese soft-shell turtle development. The results showed the dynamic changes of signaling pathways that regulate the CR development of Chinese soft-shell turtle. In particular, we revealed the Wnt signaling pathway is an important signaling pathway that regulates CR development in turtles. Interestingly, we showed that the PCP signaling pathway was activated during CR development, and *Wnt5a* participates in CR development through the JNK pathway.

The Wnt signaling pathway is a highly conserved pathway that is involved in establishing planar cell polarity in eukaryotic organisms, and in determining cell-fate, include the proliferation and migration of cells [34]. One of two subfamilies of Wnt family proteins is the Wnt1 class, which includes Wnt1, Wnt3a, and Wnt8, initiating the canonical Wnt/β-catenin pathway to play roles in cell fate and cell proliferation [34]. The other is the *Wnt5a* class, containing *Wnt5a* and Wnt11 signals, which regulate convergent extension movements in embryonic development, mainly through the PCP pathway [35]. To reveal the mechanism of the turtle carapace formation, previous studies were performed on different turtle species, including hard-shelled turtles and soft-shelled turtles, pleurodiran, and cryptodiran, which suggested that the turtle shell probably co-opted the canonical Wnt signaling pathway into the CR [6,20]. Indeed, a previous study screened CR-specific expression and found that the components of the canonical Wnt signaling pathway, Lef1 and APCDD1, were expressed in the CR mesenchyme of soft-shell turtle, and the nuclear localization of the β-catenin protein in the epithelium of CR [5]. Moreover, Nagashima used electroporation to form a dominant-negative mutant of *Lef-1*, which resulted in the local growth arrest of the CR, suggesting that Lef1 is an essential factor in the growth and maintenance of the CR [3]. Our results showed that β-catenin was not differentially expressed at the transcription and translation levels; however, several inhibitory factors were differentially expressed during CR development (Figure 7). For example, *Wif-1* was significantly differentially expressed during CR development, which was one of the important antagonists of the Wnt/β-catenin signal pathway [34,36,37,38]. *DKK* was significantly differentially expressed during CR development, which is a secreted glycoprotein that plays a negative regulatory role in the Wnt signaling pathway [39]. In addition, we observed the significant differential expression during the CR development of GSK-3β, Axin, and APC, which form a complex with β-catenin that reduces the level of free β-catenin in cells and inhibits the Wnt/β-catenin signal pathway [40]. These results suggested that the canonical Wnt signaling pathway might be inhibited during CR development. By contrast, we found that *Lef1* was differentially expressed at both the transcription and translation levels among the three comparative groups. The possible reason for this divergence is that β-catenin regulates CR development not through transcriptional regulation and translational regulation, but through its transfer to the nucleus, where it perform its functions.

Embryonic morphogenesis in complicated organisms requires patterning and directional growth under appropriate regulations [22]. PCP signaling is a crucial evolutionarily conserved mechanism that conveys directional information [24]. PCP originally referred to the coordinated alignment of epithelial cells within a perpendicular direction to their apical-basal axis as a necessary mechanism underlyingly the regulation of polarized cellular and tissue behavior [40]. This is required in left–right patterning, sensory hair cell orientation in the inner ear, skin hair orientation, and neural tube closure [41,42]. The first molecular evidence of PCP establishment is the Drosophila core PCP proteins, Frizzled, Disheveled, Van Gogh, Prickle, and Flamingo distribute asymmetrically throughout the polarized tissue [43]. Cell adhesion gradients, morphogenetic forces, and Wnt signaling gradients were suggested as mechanisms that regulate PCP establishment by global cues [22]. Wnt ligands are required to regulate PCP in vertebrates. For example, the core PCP protein, Vangl2, genetically interacts with *Wnt5a* in multiple developmental processes [44]. A putative receptor of *Wnt5a*, *Ror2*, together with *Wnt5a*, establishes PCP in Drosophila and the equivalent convergent extension movements by activating on the non-canonical Wnt signaling cascade during gastrulation in vertebrates [45,46]. Morphogen gradients involved in pattern formation are well known, and in PCP regulation, *Wnt5a* signaling is essential [47]. The non-canonical Wnt5a/JNK pathway, conserved in both vertebrates and invertebrates, activates JNK to regulate morphogenetic cell movements [45]. C-Jun is phosphorylated by MAPK-type enzymes such as JNKs and ERKs [48], which phosphorylate c-Jun by MAPK on Ser^63^ and Ser^73^, on Thr^91^ or Thr^93^, or on both to increase its trans-activating potential and DNA-binding activity (reviewed in [49]). C-Jun amino-terminal kinases (JNKs) are closely related to mitogen-activated protein kinases (MAPKs); however, they can be regulated by various environmental stimuli [50]. SAPKs were independently identified and named JNKs because of their ability to phosphorylate the amino terminus of the c-jun transcription factor [25], which is supposed to be essential for phosphorylating the c-jun protein’s trans-activating domain in vivo. Thus, phosphorylated c-jun homodimers can regulate the expression of genes, including those encoding c-jun itself, via its potent AP-1 activity [50]. In addition, JNKs link to cell surface receptors via an integral part of the small GTP-binding proteins Rac1 and Cdc42 [48]. *DVL* was originally discovered in *Drosophila* as a segment polarity gene (dsh). A previous study showed the Disheveled discriminates among canonical Wnt signaling pathways and the non-canonical Wnt signaling pathway by distinct domain interactions [25], in which the C-terminal DEP domain required for Disheveled to activate JNK cascades and induce JNK signaling [25]. Interesting, this study showed that the PCP pathway was activated during CR development. *Wnt5a*, *Wnt11*, *Frizzled*, and *Daam1* in the PCP pathway were differentially expressed among the three comparison groups. Moreover, *Dvl*, *Rac* and *JNK* in the PCR pathway were differentially expressed at the translation level among the three comparison groups. Furthermore, we knocked downed the expression of *Wnt5a* in vitro, which reduced the expression of *Dvl1*, *Ror2*, *Mapk8*, *Mapk9*, and *Damm1* in CR tissues cultured in vitro. Therefore, we concluded that *Wnt5a* may regulate CR development in Chinese soft-shell turtle through the Wnt5a/JNK pathway.

The CR structure comprises mesenchymal cells encased in an overlying thickened layer of epithelial cells, which has a similar morphological structure to the primordium of the vertebrate limb [51]. Ectodermal thickening at the distal aspect of the limb mediates proximodistal growth, which is termed the apical ectodermal ridge (AER) [52]. Cells in the mesenchyme are exposed to FGFs from the AER, in addition to Wnts from the ectoderm and AER [53,54]. The interaction of the mesenchymal factor FGF10 with FGF8 is responsible for the initiation and maintenance of chick limb bud outgrowth [55]. The combination of Wnt and FGF signals interplay to maintain the undifferentiated multipotent state, and then intensively promote limb progenitor cell growth [56,57,58]. In addition, Wnt3a and Fgf8 signals combine to retain the ability to undergo the chondrogenesis of limb progenitor cells [58]. Moreover, Wnt3a and Fgf8 promote the proliferation of limb mesenchyme and enhance the proliferative effect, respectively [58]. Wnt signals control progenitor cell segregation into chondrogenic and connective tissue lineages, while the limb ectoderm regulates various models by inhibiting chondrogenic differentiation, wherein chondrogenic cells only appear away from the range of the inhibitory signal of the ectoderm. Therefore, both the size of the limb bud and the range of the inhibitory signals are controlled by the size and location of the chondrogenic core [57,59]. In addition, the non-canonical *Wnt5a* pathway is required for limb morphogenesis [47]. *Wnt5a* does not affect axis development but causes alterations of morphogenetic cell movements [41]. Fgfs associate with limb patterning in a *Wnt5a*-dependent manner to regulate PCP along the proximal–distal axis [28]. In this study, we demonstrated dynamic changes in the signaling pathways involved in CR development. In particular, the results revealed that the Wnt signaling pathway and MAPK signaling pathway have important roles in turtle CR development. Moreover, we also showed that *Wnt5a* and *Fgf10* are specifically expressed in the CR. Therefore, we speculated that the CR is the central signaling tissue of Wnt and FGF signaling, which, through the CR mesenchyme, maintains all cells in a multipotent and proliferative state. Burke named and characterized the CR, which shows proliferation patterns and a morphogenetic molecule distribution that is similar to other organs (e.g., feathers and limbs) that develop via epithelial–mesenchymal transition [17]. The preferred hypothesis is that the CR and vertebrate limb buds have a similar morphogenesis and mechanism of their induction and growth, which suggested that these primordial tissues may also show similarities in gene expression. Therefore, we believe that the development of carapace morphogenesis acts through a co-option of morphogenetic mechanisms and gene regulatory networks, and there is also a cell lineage-related conservation of gene expression. Here, we hypothesized that once the CR is formed, the cartilaginous elements are formed according to positional information established by signaling centers of the CR, including the canonical Wnt pathway, the Wnt5a/JNK pathway and FGFs, which are coordinated to regulate the body plan of the turtle carapace. Further studies are required to confirm that WNT and FGF together regulate the development of CR.

In summary, we showed dynamic signaling pathways acting on the CR development of Chinese soft-shell turtle and revealed that the Wnt signaling pathway and MAPK signaling pathway play important roles in CR development. In addition, we found Wnt and Fgf signaling are active during the CR development of Chinese soft-shell turtle. Moreover, we confirmed that β-catenin may regulate CR development through its transfer to the nucleus and found that *Wnt5a* regulates CR development through the Wnt5a/JNK pathway. Therefore, we speculated that the morphogenesis of the turtle carapace might proceed through the co-option of the conserved canonical Wnt pathway, the Wnt5a/JNK pathway and FGFs, which are coordinated to regulate the body plan of the turtle carapace. Therefore, our findings are of great scientific significance to uncover the molecular mechanism underlying turtle carapace morphogenesis.

## 4. Materials and Methods

### 4.1. Embryo Culture and Tissue Collection

Fertilized embryos of Chinese soft-shell turtle were collected from a turtle farm in Anhui, China. Turtle eggs were incubated in an incubator at 30 ± 2 °C and a humidity of 70–80%. Embryos were staged as described by Tokita and Kuratani, abbreviated as the TK stage [60]. The 14th stage (TK14), 15th stage (TK15), and 16th stage (TK16) embryos of Chinese soft-shell turtle were washed with sterile water and then sterilized with 75% alcohol. The shells of the turtle eggs were opened using forceps, and the CR tissue was excised from the embryos using forceps under the microscope. The collected CR tissues from 25 embryos were mixed into a cryopreservation tube as one sample, and 7 samples were taken at each stage. All samples were stored at −80 °C. The TK13-TK17 embryos of Chinese soft-shell turtle were fixed using 4% paraformaldehyde (PFA) and dehydrated stepwise into 100% methanol for WISH [61]. All animal experiments in this research were performed according to the guidelines established by Anhui Agricultural University, and the experimental protocols were approved by the Animal Care and Use Committee of Anhui Agricultural University.

### 4.2. Total RNA, RNC-mRNA, Library Construction, and Sequencing

Total RNA was extracted using the Trizol reagent (Invitrogen, Waltham, MA, USA) as previously described [62]. After the total RNA was extracted, eukaryotic mRNA was enriched using Oligo (dT) beads. Then, the enriched mRNA was fragmented into short fragments using a fragmentation buffer and reverse transcribed into cDNA using random primers. Second-strand cDNA was synthesized using DNA polymerase I, RNase H, dNTP, and buffer. Then, the cDNA fragments were purified using a QiaQuick PCR extraction kit (Qiagen, Hilden, Germany), end repaired, poly(A) added, and ligated to Illumina sequencing adapters. The ligation products were size selected using agarose gel electrophoresis, PCR amplified, and sequenced using the Illumina Nova-seq system (Illumina, San Diego, CA, USA) [63].

Ribosome nascent-chain complex (RNC) extraction was performed as described by Esposito et al. [32] with certain modifications. After total RNC-RNA (translating mRNA) was extracted, eukaryotic mRNA was enriched using Oligo (dT) beads, and the enriched mRNA was fragmented into short fragments (~300) using fragmentation buffer and reverse transcribed into cDNA using random primers. Second-strand cDNA was synthesized using DNA polymerase I, RNase H, dNTP, and buffer. Then, the cDNA fragments were purified using a QiaQuick PCR extraction kit, end repaired, poly(A) added, and ligated to Illumina sequencing adapters. The ligation products were size selected using agarose gel electrophoresis, and PCR amplified. After the libraries for total RNC-RNA were successfully constructed, sequencing using Illumina HiSeq 2500 system was performed [64].

### 4.3. Transcriptome and Translatome Assembly

To obtain high-quality clean reads, reads were further filtered according to previous described rules [62]. The short reads alignment tool, Bowtie2 [65], was used to map reads to the ribosome RNA (rRNA) database. The rRNA mapped reads were then removed. The remaining reads were further used in the assembly and analysis of the transcriptome and translatome. The rRNA removed reads of each sample from RNA-seq, and RNC-seq were then mapped to the reference genome (PelSin_1.0, NCBI) by TopHat2 (version 2.0.3.12; [66,67]), respectively. The alignment parameters were as previously described [62]. After alignment with the reference genome, unmapped reads (or those that were mapped very poorly) were then re-aligned using Bowtie2, and the enriched unmapped reads were then split into smaller segments which were used to find potential splice sites. The section and the section position of these short segments were also predicted. A set of splice sites were built using the initial unmapped reads and TopHat2, without relying on the known gene annotation [68]. Not only was the sequence alignment used to identify expressed genes and their quantitative expression, it was also for finding alternative splicing and new transcripts. The reconstruction of transcripts was carried out using the software, Cufflinks [69], which, together with TopHat2, allows biologists to identify new genes and new splice variants of known ones. Cufflinks constructed faux reads according to the reference to make up for the influence of low-coverage sequencing. During the last step of assembly, all of the reassembled fragments were aligned with reference genes and then similar fragments were removed.

### 4.4. Quantification of Gene Abundance

Gene abundances were quantified using the software RSEM [70]. RSEM uses two steps to quantify gene abundance. First, a set of reference transcript sequences are generated and preprocessed according to known transcripts and new transcripts (in the FASTA format), and gene annotation files (in the GTF format). Second, RNA-seq reads are realigned to the reference transcripts using the Bowtie alignment program and the resulting alignments were used to estimate gene abundance.

The gene expression level was normalized by using the FPKM (Fragments PerKilobase of transcript per Million mapped reads) method, as previous described [71]. The FPKM method is able to eliminate the influence of different gene lengths and the amount of sequencing data from the calculation of gene expression. Therefore, the calculated gene expression can be used directly to compare the difference of gene expression among samples.

### 4.5. Differentially Transcribed Genes (DTRGs) and Differentially Translated Genes (DTLGs)

To identify DTRGs across groups, the edge R package (http://www.r-project.org/, version 3.5.2, accessed on 5 June 2021) was used. We identified genes with a fold change ≥ 2 and a false discovery rate (FDR) <0.05 in a comparison as significant DTRGs. The DTRGs from the RNA-seq samples were identified according to the different expression levels of their mRNA [62].

To search for genes with differential translation levels across groups, the translation ratio (TR = RNC-mRNA/mRNA) was calculated between each sample from the RNCseq data, and the different translation level of genes between experiment groups were identified using the software (edgeRpackage (http://www.r-project.org/, version 3.5.2, accessed on 5 June 2021)). We identified genes with a TR ≥ 2 and an FDR < 0.05 in a comparison as significant DTLGs [72].

### 4.6. Pathway Enrichment Analysis

Pathway-based analysis helped to further understand the biological functions associated with genes. The Kyoto Encyclopedia of Genes and Genomes (KEGG) is the major public pathway-related database [73]. Pathway enrichment analysis identified significantly enriched metabolic pathways or signal transduction pathways associated with DTRGs or DTLGs in comparison with the whole genome background. The calculating formula is the same as that used in gene ontology (GO) analysis [74]. The calculated *p*-value was subjected to FDR correction, using FDR ≤ 0.05 as a threshold. Pathways meeting this condition were defined as significantly enriched pathways in DTRGs or DTLGs [75].

### 4.7. Whole Mount In Situ Hybridization (WISH)

Probes were constructed as described previously [76], and the detailed experimental procedures of WISH were the same as those described in previous reports [61].

### 4.8. In Vitro Culture of Carapacial Ridge Tissues

The eggshells of TK14 embryos were cracked open with sterile tweezers, the embryos were placed into sterile phosphate-buffered saline (PBS) using the tweezers, and the extra embryonic membranes were removed. Embryos were transferred to Dulbecco’s Modified Eagle Medium (DMEM, Gibco, Grand Island, NY, USA) with 10% Penicillin (Gibco) and 10% streptomycin (Gibco) for temporary storage. The CR was excised from the embryos using forceps under the microscope, separated into small pieces by tweezers. Finally, the small pieces were added to 96-well plates containing 100 μL DMEM medium, supplemented with 2% fetal calf serum (FCS, Mediatech Inc., Manassas, VA, USA), 1% Penicillin (Gibco) and 1% streptomycin (Gibco) and cultured at 30 °C with 5% CO_2_ for 24 h.

### 4.9. RNAi Interference

CR tissues were inoculated in 96-well plates, cultured in a CO_2_ incubator, and then transfected with siRNAs after 24 h. siRNAs targeting *Wnt5a* were synthesized by Gene Pharma (Shanghai, China) with the sequence of si*Wnt5a* CAAGTGAACAGCCGTTTCA. To increase the stability of si*Wnt5a*, we modified the 5′-end with cholesterol (Cho) at Gene Pharma, to produce 5′ Cho-si-*Wnt5a*. The knock down efficiency of 5′ Cho-si-*Wnt5a* in CR tissues was detected using quantitative real-time reverse transcription PCR (qRT-PCR) after 48 h using primers for *Wnt5a* (shown in Table 1). Finally, the expression levels of *Dvl1*, *Ror2*, *Daam1*, *Mapk8*, and *Mapk9* were detected by qRT-PCR after *Wnt5a* was knocked down in CR tissues by 5′ Cho-si-*Wnt5a* transfection [77].

### 4.10. Quantitative RT-PCR

Total RNA of CR tissues were extracted using an RNA extraction kit (Sangon Biotech, Shanghai, China, B511321), as previously described [78]. Primers were designed using Primer3 (https://bioinfo.ut.ee/primer3-0.4.0/, accessed on 7 October 2021; sequences are shown in Table 1) and evaluated using BLAST at NCBI. *Gapdh* (encoding glyceraldehyde-3-phosphate dehydrogenase) was used as the internal control. Each experiment was repeated three times.

### 4.11. Statistical Analyses

Statistical analyses were performed using SAS software version 9.0 (SAS, Cary, NC, USA). All data were analyzed using one-way analysis of variance (ANOVA). Homogeneity of variances was evaluated using Levene’s test followed by Student’s *t*-test. *p* <  0.05 was considered to indicate statistical significance [79].

## Figures and Tables

**Table 1:** Primers used in the present study.

Gene Name	Forward Primer	Reverse Primer
*Wnt5a*	CGATGCCCTGAAGGAGAAAT	ATGGTGGGCGTGTTGAAAC
*Dvl1*	GGATCCCACGCCTAGAAGTT	CCCGTCATGGCTGTGGTATGA
*Daam1*	GGCTGCTAGAAAATCGCTGA	TCAAGCCGTCCAGGTCGATA
*Mapk8*	GCGTGGTCATTTGTCCTACCT	TGACTTTGCCAAGGGTCACA
*Mapk9*	ACCAGCCCTTCCCAGTCGT	GTCCCGTCAGGGCATCAAT
*Ror2*	CAGCACAAGCCCCGTTAGTA	TGGGACCATTGGTCGGATC
*Lef-1*	CAAATAAGGTGCCAGTGGTGC	AGGGATGTGTGAAGGGTGTGA
*Daam2*	GGAATTCCGATTGCACCTGA	GCTTCGCAAGTTCCAGATCA
*Dkk2*	AAGAAGCGTTGCCACAGAGA	CCAGCCCATGTCCTTGCTAG
*Gapdh*	GTGCTGCCCAGAACATCATT	GGGAGTTGGAACACGGAAAG

## Data Availability

Data were deposited in the NCBI Sequence Read Archive (SRA) PRJNA742492.

## Supplementary Materials

The following are available online at https://www.mdpi.com/article/10.3390/ijms222212441/s1.

## Abbreviations

CR	Carapace ridge
KEGG	Kyoto encyclopedia of Genes and Genomes
RNC-mRNA	ribosome nascent-chain complex-mRNA
WNT	Wingless-type
PCP	planar cell polarity
FGF	fibroblast growth factor
JNK	c-Jun N-terminal kinase
