# Supplementary figures and images for: Global Analysis of Transcriptome and Translatome Revealed That Coordinated WNT and FGF Regulate the Carapacial Ridge Development of Chinese Soft-Shell Turtle
Source: Int J Mol Sci. 2021 Nov 18;22(22):12441. doi: 10.3390/ijms222212441 (PMC8621500; doi:10.3390/ijms222212441)

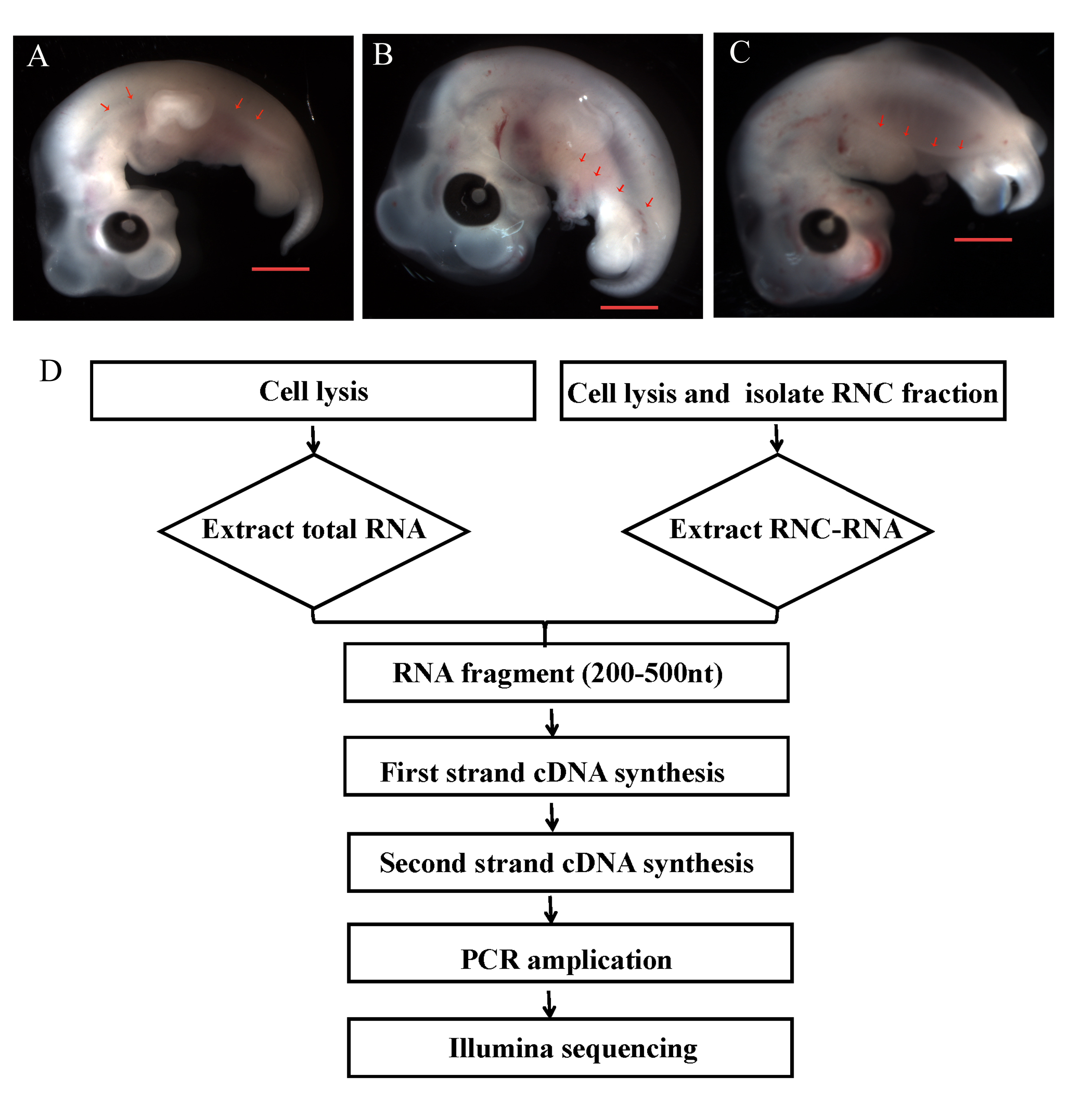

Supplement: Supplementary file 1 [file ijms-22-12441-s001.zip › Figure S1.tif]
